# A head-to-head comparison of EQ-5D-5 L and SF-6D in Chinese patients with low back pain

**DOI:** 10.1186/s12955-019-1137-6

**Published:** 2019-04-11

**Authors:** Ziping Ye, Lihua Sun, Qi Wang

**Affiliations:** 1 College of Business Administration, Shenyang Pharmaceutical University, Shenyang, China; 2 Department of orthopedics, The General Hospital of Shenyang Military Area Command, Shenyang, China

**Keywords:** EQ-5D-5 L, SF-6D, The Oswestry questionnaire, Low back pain

## Abstract

**Background:**

The comparative performance of the 3-level EuroQol 5-dimension and Short Form 6-dimension (SF-6D) has been investigated in patients with low back pain (LBP). The aim of this study was to explore the performance including agreement, convergent validity as well as known-groups validity of the 5-level EuroQol 5-dimension (EQ-5D-5 L) and SF-6D in Chinese patients with LBP.

**Methods:**

Individuals with LBP were recruited from a large tertiary hospital in China. All subjects were interviewed using a standardized questionnaire including the EQ-5D-5 L, 36-item Short Form Health Survey (SF-36), the Oswestry questionnaire and socio-demographic questions from June 2017 to October 2017. Agreement was evaluated by intra-class correlation coefficients (ICCs) and Bland–Altman plots. Spearman’s rank correlation coefficients were applied to assess convergent validity. For known-groups validity, the Mann–Whitney U test or Kruskal-Wallis H test were used, effect size (ES) and relative efficiency (RE) were also reported. The efficiency of detecting clinically relevant differences was measured by receiver operating characteristic (ROC) curves between pre-specified groups based on Oswestry disability index (ODI), ES and RE statistics were also reported.

**Results:**

Two hundred seventy-two LBP patients (age 38.1, 38% female) took part in the study. Agreement between the EQ-5D-5 L and the SF-6D was good (ICC 0.661) but with systematic discrepancy in the Bland–Altman plots. In terms of convergent validity, most priori assumptions were more related to EQ-5D-5 L than SF-6D, but MCS derived from SF-36 was more associated with SF-6D. EQ-5D-5 L demonstrated better performance for most groups except location and general health grouped by the general assessment of health item from SF-36. Furthermore, when we applied ODI as external indicator of health status, the area under the ROC curve for EQ-5D-5 L was larger than that for the SF-6D (0.892, 95% CI 0.853 to 0.931 versus 0.822, 95% CI 0.771 to 0.873), the effect size was 0.63 for EQ-5D-5 L and 0.44 for SF-6D, and it was proved that EQ-5D-5 L was 42% more efficient than SF-6D at detecting differences measured by ODI.

**Conclusions:**

Both EQ-5D-5 L and SF-6D are valid measures for LBP patients. Even though these two measures had good agreement, they cannot be used interchangeably. The EQ-5D-5 L was superior to the SF-6D in Chinese low back pain patients in this research, with stronger correlation to ODI and better known-groups validity. Further study needs to evaluate other factors, such as responsiveness and reliability.

## Background

Low back pain (LBP) is a common condition that can cause severe activity impairment and physical limitations [1]. Among employees of China, the prevalence of LBP is around 42.7–72.0%, which makes LBP the most common cause of physical disability [2, 3]. As an incapacitating disease, LBP is related to significant reduction in health-related quality of life (HRQoL) [4]. Hence, a valid and reliable HRQoL measure is needed to evaluate interventions or programs for LBP, and inform resource allocation decisions.

In general, HRQoL can be assessed using either disease-specific or generic instruments. The generic instruments can be in turn subdivided into: preference-based and non-preference based. The main benefit of generic preference-based measures is their broad range of health dimensions, which makes the comparisons of various disease, interventions and health programs possible [5]. Besides, generic preference-based measures provide a general estimate of health outcomes and can capture survival data in the form of quality-adjusted life years (QALYs), which is largely used as clinical effectiveness indicator [6].

The EuroQol 5-dimension (EQ-5D) is the most frequently used preference-based instrument around world [7]. Due to the high ceiling effects of the three level of EQ-5D (EQ-5D-3 L), a new version of the EQ-5D (known as EQ-5D-5 L) was developed [8]. With increasing availability of national value sets, crosswalk algorithms for converting 3 L scores to 5 L scores and more evidence about better psychometric properties of EQ-5D-5 L, we could observed increased uptake of the EQ-5D-5 L. Since Luo and colleagues [9] developed the scoring algorithm for the EQ-5D-5 L based on Chinese preference, the EQ-5D-5 L is becoming popular in clinical studies in China. The Short Form 6-dimension (SF-6D) is a utility measure from the 36-item Short Form Health Survey (SF-36) [10], which has been considered as one of the most widely used generic measures of HRQoL in clinical trials. A number of studies have explored the performance of EQ-5D and SF-6D in various patient sets, and the results showed that comparative validity and responsiveness differed depending on the target population [11–14].

The comparative performance of the EQ-5D and SF-6D has been investigated in patients with LBP [15, 16], and it was found that EQ-5D and SF-6D were not interchangeable with the SF-6D largely outperforming the former in terms of measurement characteristics. However, both studies applied the 3-level version of the EQ-5D (EQ-5D-3 L), which was found to possess poor discriminative ability [17] and ceiling effects [18]. Several studies found better psychometric properties for the EQ-5D-5 L compared with EQ-5D-3 L [19–22]. Therefore, it seems vital to compare the EQ-5D-5 L with SF-6D in LBP patients. Hence, this study attempts to evaluate agreement, convergent validity as well as known-groups of EQ-5D-5 L and SF-6D in patients with LBP.

## Methods

### Study design and patient recruitment

After being approved by ethics committee, consecutive patients of this cross-sectional study were recruited at the General Hospital of Shenyang Military Area Command in Shenyang city of China from June 2017 to October 2017. The inclusion and exclusion criteria were as follows.

Inclusion criteria: Patients with LBP aged more than 18, with or without the lower limb pain, not experiencing any other coexisting treatments for pain except routine painkilling, understanding and speaking Mandarin; Exclusion criteria: patients with coexisting infection, malignancy, severe spinal cord disease or inflammatory joint disease; patients with myocardial infarction, cerebrovascular events, chronic lung disease, kidney disease or severe mental illness; pregnant women.

Confidence intervals were used to estimate the sample mean using following equation [23]:

$$ \mathrm{n}=\frac{\sigma^2}{{\left[\frac{\omega }{1.96}\right]}^2} $$

ω is the margin of error, σ is the outcome variable standard deviation (assumed to be the same under the null and alternative hypotheses). We wish ω to be 0.03 for all measures, σ = 0.238 for EQ-5D-5 L [24], σ = 0.152 for SF-6D [15], σ = 0.2026 for ODI [15], which gives an estimated sample size for the survey of *n* = 242, 98 and 176 for EQ-5D-5 L, SF-6D and ODI respectively. Assuming an 80% response rate to the survey, we would like to interview 300 LBP patients.

The diagnosis of LBP was based on the imaging information, physical examination as well as patients’ complaints of LBP. As all the questionnaires used in this survey were verified, no pilot or pre-testing survey was performed. After submitting formal consent, every patient was questioned by the same interviewer. The interviewer was trained to conduct the survey in the same manner. At outpatient clinics, individuals were interviewed in the waiting room after consultation; at inpatient clinics, the survey was implemented in the sickroom before operation. The questions of the survey were organized in the following order: socio-demographic queries, Oswestry disability questionnaire, questions regarding the EQ-5D-5 L and SF-36. The interviewer, procedure, and questionnaire were the same for all patients.

### Instruments and measures

#### EQ-5D-5 l

The EQ-5D-5 L contains two parts that assesses health status of respondents on the day of interview [8]. The first part is a descriptive system with five items (mobility, self-care, pain/discomfort, usual activities, and anxiety/depression), every item has five different levels of severity. Theoretically, the EQ-5D-5 L can define 3125 different health states. In accordance with the Chinese scoring algorithm [9], the EQ-5D-5 L gives a score from − 0.39 to 1 where 1 is the best possible health state. The other part of EQ-5D-5 L is a visual analogue scale (EQ-VAS), asking interviewees to mark their present health status on a 20 cm vertical scale from 0 to 100. The simplified Chinese version of EQ-5D-5 L in our research is approved by the EuroQol Group.

#### SF-36 based SF-6D

The SF-6D is an utility measure which was derived from the SF-36 [10]. Health status here is defined in terms of 6 dimensions (physical functioning, role limitation, social functioning, pain, energy and mental health), with each dimension having four to six levels. There are potentially 18,000 different health states. A value set for general population in Hong Kong [25] was used to estimate utility index for the SF-6D in this study. Utility score of SF-6D can range from 0.315 to 1.00. As recommended by previously published research [26], SF-36v2 was used as questionnaire when the survey was conducted instead of applying SF-6D as an independent instrument. The official version of SF-36 in simplified Chinese was authorized by QualityMetric [27].

#### Oswestry disability index

The Oswestry Disability Index (ODI) [28, 29] is an instrument measuring degree of disability in people with LBP. This questionnaire contains 10 items, including intensity of pain, personal care, lifting, walking, sitting, standing, sleeping, sex life, social life, and traveling. Each item is followed by 6 different levels, with scores from 0 (the least disability) to 5 (the most severe disability). The sum of all item scores is needed to transform into a 0 to 100% index. Patients with scores between 0 and 20% have minimal disability, 21 to 40% moderate disability, 41 to 60% severe disability, 61 to 80% unable to walk which was always defined as crippled, and 81 to 100% [30] bedbound or overstating their symptoms. Previous studies found the item about “sex life” culturally inappropriate for Chinese citizens [31]. Hence, we applied only 9 items in the ODI. The Chinese version of the ODI was an official version from Mapi Research Trust.

### Statistical analysis

#### Patient characteristics and descriptive statistics

Only patients who completed all questionnaires were included in this analysis, we did not perform further imputation for missing scores. Continuous variables were reported as means and standard deviations (SD), frequencies and proportions were used for categorical variables. Descriptive statistics (mean, SD, median, inter-quartile range, minimum and maximum) for the ODI, EQ-5D-5 L and SF-6D were computed. Floor and ceiling effects for EQ-5D-5 L and SF-6D were evaluated by calculating the proportion of sample in the worst and best possible health states. Statistical analysis was conducted using IBM SPSS version 23.0 [32].

#### Agreement between the EQ-5D-5 L and SF-6D

When we repeat measurements by each of two methods on the same subjects, agreement analysis is essential to see whether they agree sufficiently for one method to replace the other one [33]. Both EQ-5D-5 L and SF-6D are measures for health utility, even though the EQ-5D-5 L has a possible range of − 0.39 to 1.00, while the SF-6D has a range of 0.315 to 1.00. Hence, it is necessary for us to know to what degree these two utility measures agree and if it is possible to use these two measures interchangeably in the context of LBP patients in China. Agreement was assessed by intra-class correlation coefficients (ICCs) and Bland-Altman plots. The ICCs were calculated with two-way random effects model using average measures and absolute agreement. The ICCs can range between 0 and 1. An ICC < 0.4 suggests poor agreement, 0.4–0.59 fair, 0.6–0.74 good, and 0.75–1 excellent agreement [34]. Bland-Altman plots were also performed to explore the agreement between these two measures. In this method, the differences between the scores of the two instruments were plotted against the average utility scores [35].

#### Convergent validity

Following previous research [12, 36–38], the size of the correlations was compared for the EQ-5D-5 L and SF-6D scores with the ODI, the EQ-VAS, SF-36 physical (PCS) and mental component summary (MCS). The association was evaluated by Spearman’s rank correlation coefficient, considering 0.9–1.0 as very highly correlated, 0.7–0.9 as highly correlated, 0.5–0.7 as moderately correlated, and 0.3–0.5 as low correlated [39].

#### Known-groups validity

##### General known-group validity

EQ-5D-5 L and SF-6D scores were compared across important groups. Therefore, we divided sample by demographic characteristics, duration of pain [40], outpatients and inpatients, the general assessment of health item from SF-36 and EQ-VAS. It was hypothesized that patients with lower utility scores included the elderly [41], females [41], patients with longer duration of disease [36] and lower education [42], patients from rural areas [43] and with lower income [44], even though U-shaped relationships between income and health status were reported in some studies [45].

Age was divided into two groups based on medians [36]. Education level was regrouped into three sub-levels, <=junior school, high school as well as > = college. Income data was divided into four categories: <1000yuan, 1001–3500yuan, 3501–5000 yuan and > 5000 yuan. We categorized the EQ-VAS scores into four groups, with score < 65 as bad health, 65–79 as fair health, 80–89 as good health, and 90–100 as excellent health [46]. To investigate whether dichotomous variables had significant impact on utility scores, Mann-Whitney U-tests were implemented [47]. For polychromous variables, Kruskal-Wallis H tests were used. The effect size (ES) and relative efficiency (RE) statistics were also applied. The ES was calculated using the statistics from above-mentioned tests, which was recommended by a recent published review [48], indicating the percentage of variance in the dependent variable explained by the independent variable. The RE was based on the ratio of statistics from the Mann–Whitney U or Kruskal-Wallis H tests on the EQ-5D-5 L and the SF-6D. The statistic of the SF-6D was the reference. Thus, if the RE was higher than 1, the EQ-5D-5 L was believed to be more efficient for discriminating between known groups than SF-6D.

##### Efficiency of detecting clinically relevant differences measured by ODI

The efficiency of the EQ-5D-5 L and SF-6D to distinguish clinical relevant change of individuals with LBP was measured using the ES, RE, and receiver operating characteristic (ROC) curves. The utility instrument that creates the largest area under the ROC curve is considered to be the most sensitive measure at detecting differences of external indicator. An area under the curve (AUC) of 1 denotes perfect sensitivity, whereas an area of 0.5 represents less efficient [49]. ODI was applied as external indicator, which classified individuals into five different groups. For more valid outcomes [50], ODI was also dichotomized using different cut-off points.

## Results

### Patient characteristics and descriptive statistics of ODI, EQ-5D-5 L and SF-6D utility scores

Two hundred seventy-two patients out of 300 (total number of patients who participated in the survey) were included in the research, thus we achieved 91% response rate. 28 individuals were not included in this research for the following reasons: not completing the questionnaires (*N* = 17) or being too young/too old for the research (*N* = 11).

Demographic and clinical characteristics are presented in Table 1. The mean age of participants was 38.1 years and the proportion of female was 38%. 69% of sample was from urban population. About 40% of the patients had education of college. Around 28% of patients had income of 1001–3500 Yuan. Most patients had suffered LBP for more than 12 weeks.

**Table 1:** Patient characteristics (*N* = 272)

Characteristics	Values
Age (years)	
Mean ± SD	38.1 ± 14
Median (interquartile range)	35 (26–49)
Gender, N(%)	
Male	168(62%)
Female	104 (38%)
Education, N(%)	
≤ Primary school	26 (10%)
Secondary school	68 (25%)
High school	68 (25%)
≥ College	110 (40%)
Location, N(%)	
City	188 (69%)
Villiage	85 (31%)
Income (yuan), N(%)	
≤ 1000	58 (21%)
1001–3500	75 (28%)
3501–5000	65 (24%)
5001–10,000	61 (22%)
> 10,000	13 (5%)
Duration of LBP, N(%)	
< 4 weeks	50 (18%)
4–12 weeks	43 (16%)
> 12 weeks	179 (66%)

*LBP* Low back pain, *SD* Standard deviation

**Fig. 1:**
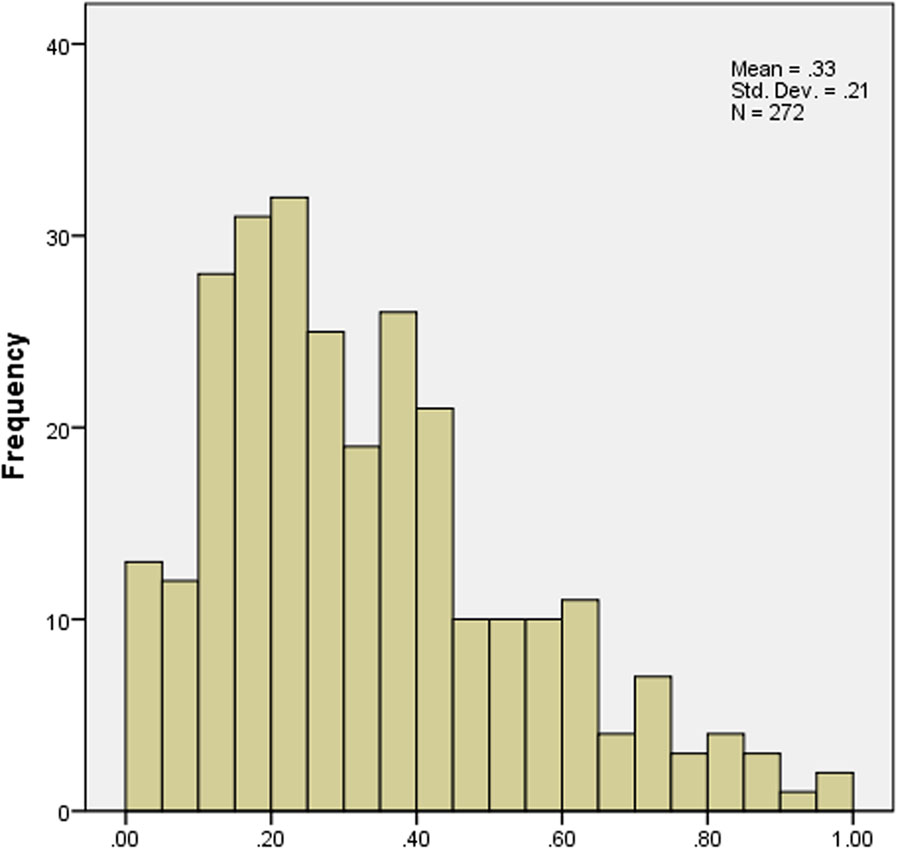
The distribution of values for the ODI

**Fig. 2:**
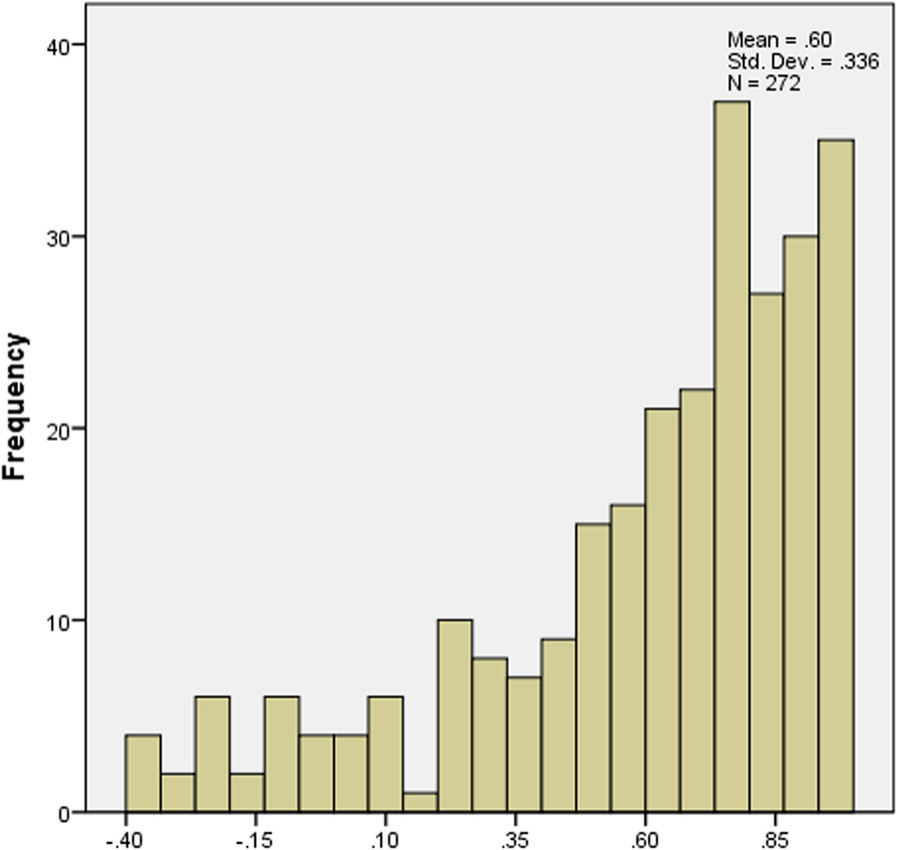
The distribution of values for the EQ-5D-5 L

**Fig. 3:**
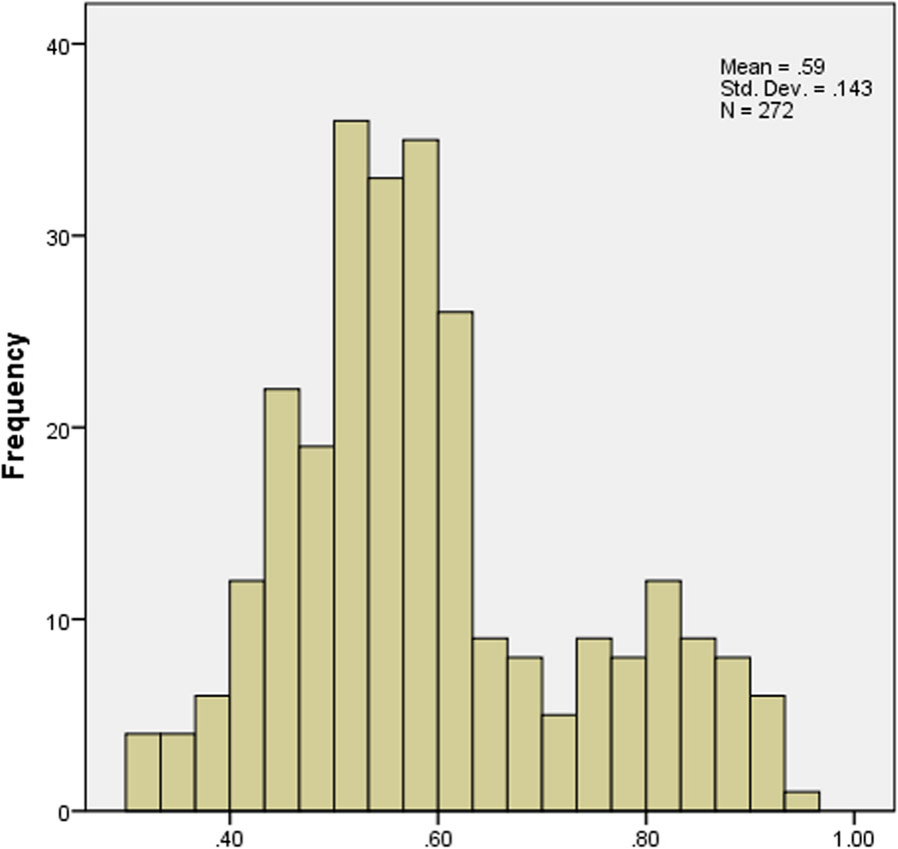
The distribution of values for the SF-6D

The distribution of scores for the ODI, EQ-5D-5 L and SF-6D is displayed in Figs. 1, 2 and 3. Moreover, descriptive statistics of the ODI, EQ-5D-5 L and SF-6D index are demonstrated in Table 2. The mean ODI was 33.1% (SD 0.210) (median 28.9%; IQR (17.8, 44.4%)), with a distribution skewed towards full health. The mean EQ-5D-5 L score was 0.603 (SD 0.336) (median 0.702; IQR (0.438, 0.862)). The distribution of EQ-5D-5 L was skewed towards full health as well, which was akin to ODI. The score range of EQ-5D-5 L was from − 0.39 to 1. The mean scores of SF-6D was 0.593 (SD 0.143) (median 0.567; IQR (0.500, 0.656)) with a distribution more symmetric around its mean. The score range of SF-6D was from 0.320 to 0.960 (Fig. 3).

**Table 2:** Descriptive statistics of ODI, EQ-5D and SF-6D utility scores, *n* = 272

	ODI	EQ-5D-5 L	SF-6D
Mean	33.1%	0.603	0.593
Standard deviation	0.210	0.336	0.143
Median	28.9%	0.702	0.567
Inter-quartile range	(17.8, 44.4%)	(0.438,0.862)	(0.500,0.656)
Maximum	98%	1.000	0.960
Minimum	0.0%	−0.390	0.320
Floor effect, N(%)	3 (1.1%)	3 (1.1%)	4 (1.5%)
Ceiling effect, N(%)	0 (0%)	1 (0.4%)	0 (0%)

*ODI* Oswestry Disability Index, *EQ-5D-5 L* 5-level EuroQol 5-dimension, *SF-6D* Short Form 6-dimension

Floor and ceiling effect were low for all three measurements. EQ-5D-5 L showed a slightly ceiling effect for 0.4% (*N* = 1), floor effect for 1.1% (*N* = 3). The ODI and SF-6D yield no ceiling effect, but indicated a small floor effect for 1.1% (*N* = 3) and 1.5% (*N* = 4) of the respondents.

### Agreement between the EQ-5D-5 L and SF-6D

**Fig. 4:**
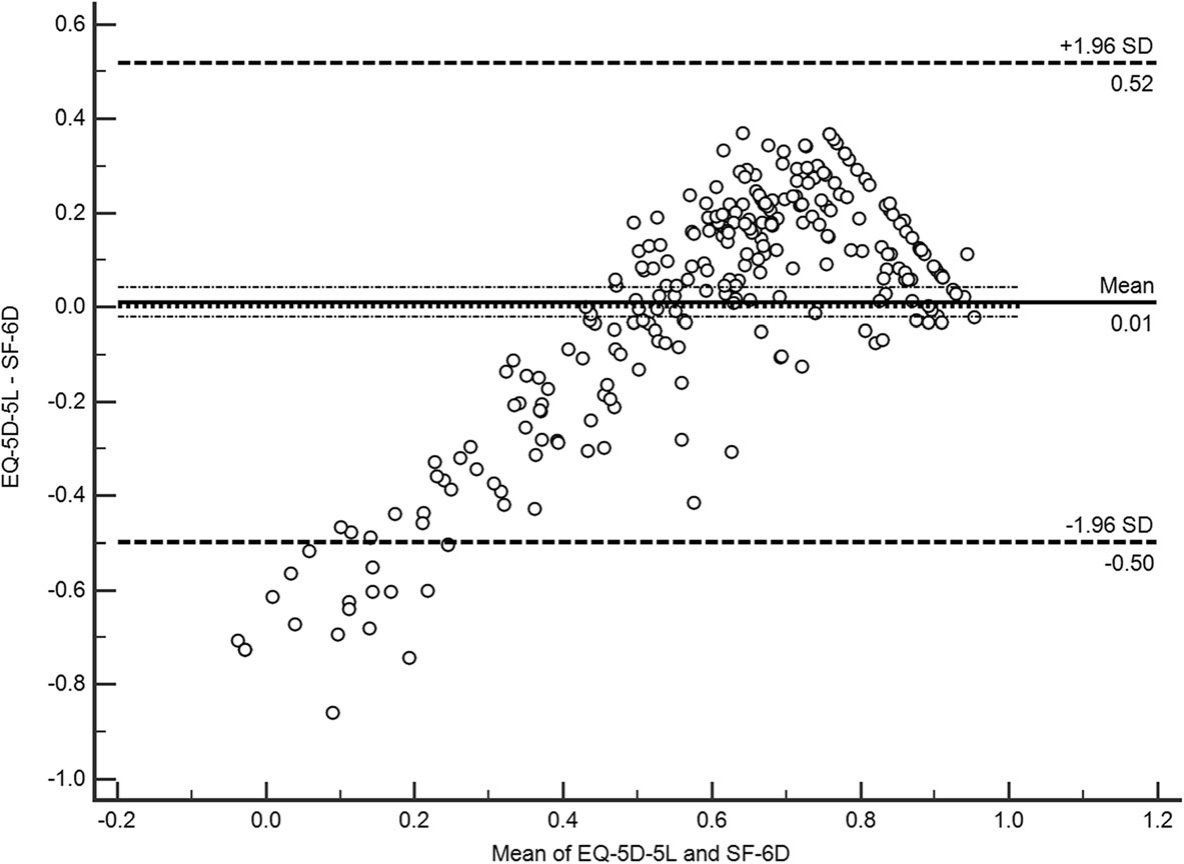
Bland–Altman plot of difference in utility scores between the EQ-5D-5 L and SF-6D

The agreement between EQ-5D-5 L and SF-6D was good, with ICC of 0.661 (95%CI 0.57–0.733). Considering the fact that ICC might be influenced by scaling differences between the EQ-5D-5 L and the SF-6D, we reanalyzed the ICC after truncating the EQ-5D-5 L index score at 0, results were similar with those without truncation. The Bland–Altman plot (Fig. 4) demonstrated a comparable picture with the ICC, as a mean difference in utility between these two measures of 0.01. The plot showed that approximately 93.8% of the utility scores were between the bounds of agreement (0.52 and − 0.50) (Fig. 4). Particularly, EQ-5D-5 L and SF-6D utility index appeared to be less consistent at the relatively bad health status where sores were outside the limit of the agreement lie. Systematic discrepancy was observed in the mean difference between these two measures, with higher SF-6D scores at low mean utility scores, and higher EQ-5D-5 L scores at high mean utility scores.

### Convergent validity

The spearman’s rho correlation coefficients were based on various a priori assumptions. Our results suggested that EQ-5D-5 L was highly associated with ODI (ρ = − 0.828), and SF-6D was moderately correlated with ODI (ρ = − 0.700). EQ-VAS was more interrelated with EQ-5D-5 L (ρ =0.544) score than SF-6D (ρ =0.472). In addition, PCS from SF-36 was highly correlated with both measures, but MCS showed week correlation with EQ-5D-5 L (ρ =0.403) but high correlation with SF-6D (ρ =0.709). All tests were proved to be statistically significant.

### Known-groups validity

#### General known-group validity

General known-groups validity is displayed in Table 3. The EQ-5D-5 L and SF-6D were both able to detect significant utility differences between outpatients and inpatients, age, education as well as income. It was found that inpatients and the elderly had lower utility scores than outpatients and the young. Monotonic relationship was observed between education levels and utility scores measured by the EQ-5D-5 L. However, using SF-6D as utility measure, those who received high school education had similar utility scores compared to those who had higher education. Nevertheless, non-monotonic relationship was noticed between income levels and utility scores for both utility measures. The comparatively low utility scores of both EQ-5D-5 L and SF-6D were observed in the very good health group (SF-36) most probably because there was only one person in this sample. However, statistically significant difference of gender, location or duration of LBP was not detected by both measures. As for education, EQ-5D-5 L showed better known-groups validity (*P* = 0.005) than SF-6D (*P* = 0.014) with RE as 1.46. The RE values of the EQ-5D-5 L were greater than 1 at discriminating patient types, gender, age, education, duration of disease and general health grouped by EQ-VAS, indicating better known-groups validity of EQ-5D-5 L. However, the EQ-5D-5 L had RE less than 1 for location and general health grouped by the general assessment of health item from SF-36.

**Table 3:** Known-group validity of EQ-5D-5 L and SF-6D

	EQ-5D-5 L			SF-6D			
Patient characteristics	Mean ± SD	P*	ES	Mean ± SD	P*	ES	RE
Patient types							
Outpatient	0.75 ± 0.21	0.000	0.360	0.64 ± 0.14	0.000	0.230	1.57
Inpatient	0.35 ± 0.35			0.51 ± 0.1			
Gender							
Male	0.62 ± 0.33	0.174	0.010	0.6 ± 0.14	0.279	0.004	1.57
Female	0.57 ± 0.34			0.58 ± 0.14			
Age							
Age < =35	0.71 ± 0.24	0.000	0.110	0.63 ± 0.14	0.000	0.070	1.45
Age > 35	0.48 ± 0.38			0.55 ± 0.14			
Location							
City	0.6 ± 0.35	0.764	0.000	0.6 ± 0.15	0.584	0.001	0.30
Village	0.61 ± 0.31			0.58 ± 0.14			
Education							
< =Junior school	0.52 ± 0.36	0.02	0.040	0.56 ± 0.13	0.013	0.030	2.01
High school	0.58 ± 0.35			0.61 ± 0.15			
> =college	0.68 ± 0.28			0.61 ± 0.14			
Income							
< 1000yuan	0.49 ± 0.36	0.000	0.1	0.54 ± 0.13	0.000	0.08	1.19
1001–3500yuan	0.51 ± 0.37			0.56 ± 0.14			
3501-5000yuan	0.74 ± 0.24			0.66 ± 0.15			
> 5000yuan	0.68 ± 0.29			0.61 ± 0.12			
Duration of disease							
< 4 Weeks	0.63 ± 0.36	0.247	0.003	0.62 ± 0.15	0.333	0.001	1.27
4–12 Weeks	0.65 ± 0.33			0.60 ± 0.14			
> 12 Weeks	0.59 ± 0.33			0.58 ± 0.14			
Genaral health (EQ-VAS)							
Bad	0.44 ± 0.37	0.000	0.220	0.54 ± 0.14	0.000	0.170	1.29
Fair	0.65 ± 0.28			0.59 ± 0.11			
Good	0.79 ± 0.19			0.65 ± 0.13			
Excellent	0.76 ± 0.25			0.68 ± 0.14			
General health (SF-36)							
Very good¶	0.49	0.000	0.230	0.56	0.000	0.250	0.91
Good	0.77 ± 0.25			0.73 ± 0.15			
Fair	0.74 ± 0.29			0.64 ± 0.13			
Bad	0.63 ± 0.27			0.58 ± 0.12			
Very bad	0.32 ± 0.38			0.49 ± 0.1			

*P is from Mann-Whitney U-tests or Kruskal-Wallis H tests (2-tailed)

¶ only one person was in this group

*EQ-5D-5 L* 5-level EuroQol 5-dimension, *SF-6D* Short Form 6-dimension, *SD* Standard deviations, *ES* Effect size, *RE* Relative efficiency, *EQ-VAS* EuroQol visual analogue scale, *SF-36* 36-item Short Form Health Survey

#### Efficiency of detecting clinically relevant differences measured by ODI

**Fig. 5:**
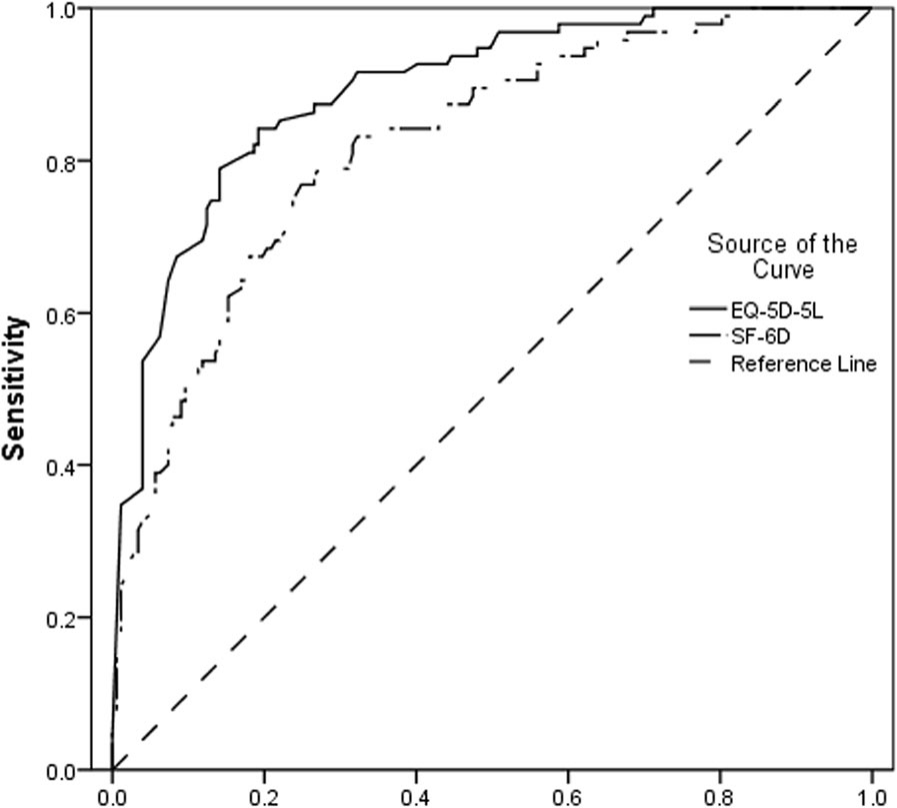
Receiver operating characteristic (ROC) curves of EQ-5D-5 L and SF-6D

The EQ-5D-5 L and SF-6D were both able to detect statistically significant utility differences depending on five categories of health states derived from the ODI (Table 4). The effect size was 0.63 for EQ-5D-5 L and 0.44 for SF-6D, and EQ-5D-5 L was found to have between 133 and 151% of the efficiency of the SF-6D at detecting differences measured by ODI. Additionally, the AUC score for EQ-5D-5 L was larger than that for the SF-6D (0.892, 95% CI 0.853 to 0.931 versus 0.822, 95% CI 0.771 to 0.873, Fig. 5). When the ODI was dichotomized into two categories by different cut-off point, the EQ-5D-5 L was proved to be between 133 to 148% efficient of the SF-6D (Table 5). For all dichotomous arrangements, the AUC scores revealed that both measures were sensitive to ODI. Moreover, the EQ-5D-5 L had higher AUC scores than SF-6D, indicating better sensitivity performance.

**Table 4:** Efficiency of EQ-5D-5 L and SF-6D to detect differences of five groups of ODI

		N (%)	EQ-5D-5 L	SF-6D
			Mean ± SD	Mean ± SD
minimal disability		95 (35%)	0.85 ± 0.13	0.7 ± 0.14
moderate disability		98 (36%)	0.67 ± 0.18	0.58 ± 0.09
severe disability		47 (17%)	0.4 ± 0.24	0.51 ± 0.1
unable to walk		24 (9%)	0.02 ± 0.25	0.44 ± 0.07
bedbound		8 (3%)	−0.23 ± 0.14	0.42 ± 0.07
ES			0.63	0.44
RE			1.42	1
ROC curve	AUC		0.892*	0.822*
	95% CI		(0.853,0.931)	(0.771,873)

*indicates that area under the ROC curve statistically significantly greater than 0.5 and that measure has discriminatory power (*P* < 0.001)

*EQ-5D-5 L* 5-level EuroQol 5-dimension, *SF-6D* Short Form 6-dimension, *ODI* Oswestry Disability Index, *SD* Standard deviations, *ES* Effect size, *RE* Relative efficiency, *ROC* Receiver Operating Characteristic, *AUC* Area Under Curve, *CI* Confidence Interval

**Table 5:** Efficiency of EQ-5D-5 L and SF-6D to detect differences of dichotomized ODI groups

Measure	Dichotomies of ODI health status†	N (%)	Mean ± SD	ES	RE	ROC curve	
						AUC	95% CI
EQ-5D-5 L	1	95 (35%)	0.85 ± 0.13	0.42	1.48	0.89*	(0.85, 0.93)
	2–5	177 (65%)	0.47 ± 0.34				
SF-6D	1	95 (35%)	0.7 ± 0.14	0.28	1	0.82*	(0.77, 0.87)
	2–5	177 (65%)	0.54 ± 0.11				
EQ-5D-5 L	1 and 2	193 (71%)	0.76 ± 0.18	0.47	1.4	0.93*	(0.90, 0.97)
	3–5	79 (29%)	0.22 ± 0.32				
SF-6D	1 and 2	193 (71%)	0.64 ± 0.13	0.33	1	0.87*	(0.82, 0.91)
	3–5	79 (29%)	0.48 ± 0.1				
EQ-5D-5 L	1–3	240 (88%)	0.69 ± 0.24	0.27	1.33	0.97*	(0.94, 0.99)
	4 and 5	32 (12%)	−0.04 ± 0.25				
SF-6D	1–3	240 (88%)	0.61 ± 0.14	0.2	1	0.91*	(0.86, 0.95)
	4 and 5	32 (12%)	0.43 ± 0.07				

†1 to 5 represents different levels of low back pain measured by ODI from minimal disability to bedbound

*indicates that area under the ROC curve statistically significantly greater than 0.5 and that measure has discriminatory power (*P* < 0.001)

*EQ-5D-5 L* 5-level EuroQol 5-dimension, *SF-6D* Short Form 6-dimension, *ODI* Oswestry Disability Index, *SD* Standard deviations, *ES* Effect size, *RE* Relative efficiency, *ROC* Receiver Operating Characteristic, *AUC* Area Under Curve, *CI* Confidence Interval

## Discussion

The purpose of this research was to compare the performance of EQ-5D-5 L and SF-6D including agreement, convergent validity and known-groups validity in patients with LBP. It was turned out that the agreement between EQ-5D-5 L and SF-6D was good. In terms of convergent validity, most priori assumptions were more associated with EQ-5D-5 L than SF-6D, but MCS derived from SF-36 was more correlated with SF-6D. As for known-groups validity, EQ-5D-5 L demonstrated better performance for most groups except location and the general assessment of health item from SF-36. Besides, EQ-5D-5 L had higher ES, RE and AUC scores when we applied ODI as external indicator of health status, which indicated that EQ-5D-5 L was more efficient at detecting clinical differences.

We found that the distributions of ODI and EQ-5D-5 L skewed towards full health. However, the distribution of SF-6D was more symmetric around its mean, reflecting previous findings [36, 51]. The distributions of these measures implied that EQ-5D-5 L might be more related to the ODI. Previously published papers declared that EQ-5D-5 L suffered high ceiling effect, which was not observed in this research [51]. One possible reason is that patients recruited in this research were from a tertiary hospital which patients visit only when they cannot endure their symptoms. In addition, unlike other diseases, LBP may drastically deteriorate quality of life [52].

The ICC of EQ-5D-5 L and SF-6D was 0.661, representing good agreement between these two measurements. This is higher than that in other similar studies in China, which is 0.448 for stable angina patients [53], 0.444 for chronic prostatitis patients [54]. Except for the fact that the study was conducted on a different disease, another possible reason for the discrepancy is that EQ-5D-3 L rather than EQ-5D-5 L were applied in these two studies. A smaller range of EQ-5D-5 L utility scores (− 0.39 to 1) was used compared with that of EQ-5D-3 L (− 0.59 to 1), which might account for the better agreement between the EQ-5D-5 L and SF-6D in this research. In consensus with previous studies in low back pain [15, 55, 56], for poorer health status, SF-6D yielded higher score, whereas EQ-5D-5 L inclined to produce higher scores for better health status. This is means that these two measures cannot be used interchangeably.

Our convergent validity analysis showed that the ODI was interrelated strongly with the EQ-5D-5 L while moderately with SF-6D. One may find this is in agreement with the previously published research [16]. The EQ-5D-5 L was more correlated with the EQ-VAS than SF-6D. A possible explanation could be that self-rated health on a VAS is a fragment of the EQ-5D-5 L, both measure the health state on the day of interview. However, a four-week recall period is used for SF-6D, which is derived from the SF-36. The fact that the SF-6D was derived from the SF-36 might show positive impact on the correlations among SF-6D and the PCS and MCS. However, the EQ-5D-5 L was more related with PCS, the SF-6D was more correlated with MCS. This is in line with previous studies from Richardson et al. [12] and Sakthong et al. [36]. Due to the fact that four of five items of the EQ-5D-5 L covers physical health, while the SF-6D entails a relatively equivalent number of physical-related items and mental-related items, one may find that the EQ-5D-5 L performs better for individuals with more physical-related health problems than those with mental-related problems [12, 36]. Given the concern that the items of ODI are more physical-related than psychological-related, this might explain that ODI correlated strongly with the EQ-5D-5 L while only moderately with SF-6D.

Both measures can discriminate patients in most known groups. EQ-5D-5 L provided higher ES and RE values for all known groups apart from location and general health grouped by the general assessment of health item from SF-36. It turned out that the outcomes of validity analysis here were in agreement with previously published studies [14, 36]. EQ-5D-5 L was 42% more efficient than SF-6D at detecting clinically relevant differences measured by ODI. Furthermore, the AUC score of EQ-5D-5 L was higher, even though there was some overlapping of 95% confidence interval between these two measures. Our study do not support the findings of Johnsen et al. [16], which concluded that SF-6D had the better ability of detecting clinical change of LBP patients than EQ-5D-3 L. Quite a few studies in various patient populations have found that EQ-5D-5 L is more discriminative than the EQ-5D-3 L [17–22, 57, 58]. Therefore, in all likelihood the increased discriminative power from the 2 additional categories is the reason for the disagreement between our research and previous study [16].

It was hypothesized that patients with higher income should have higher utility scores. Nevertheless, the estimates of utility scores of different income groups showed different results. Specifically, those who earned more than 5000 yuan had lower utility scores than those who had income between 3501 to 5000 yuan. We further analyzed the ODI for different income groups, which indicated similar tendency of health utility score. The survey was conducted at both outpatient clinics and inpatients clinics. With higher possibility to afford the operation, more severe LBP patients with high income were recruited for this research, which may explain above-mentioned issue.

The overall dissimilarity among different measures is the product of the differences in description, valuation and changes in population views of health. Since there is no comparability between the health utility scores measured by different methods [59], a rather consistent measure should be suggested. For example, EQ-5D is the only health utility measure that the National Institute of Health and Care Excellence (NICE) in England recommends. In many respects, EQ-5D-5 L is superior to the 3 L version including distributional evenness, efficiency of scale use and the face validity of the resulting distributions [60]. In many cases, EQ-5D-5 L performed better than the SF-6D. If SF-6D was used in relative clinical trial, mapping algorithm might be needed.

Obviously, there are a number of limitations to this study. Firstly, responsiveness and reliability of EQ-5D-5 L and SF-6D, which are also essential factors to choose a proper measure, were not evaluated in this study. Secondly, considering the rank of this survey is characteristic, ODI, EQ-5D-5 L and SF-36, questions in ODI may have context effect on EQ-5D-5 L, moreover, questions in ODI and EQ-5D-5 L may have context effect on SF-36. The term “context effect” refers to a process in which prior questions affect responses to later questions in surveys [61]. Thirdly, since there was no Chinese value set for SF-6D, Hong Kong value set was applied, which might influence our findings. Fourth, interview administration rather than self-completed mode of administration was applied in this research, which might influence the generalizability of the outcomes. Finally, the sample were recruited from the orthopedics outpatient and inpatient clinic from one tertiary hospital in China, hence, the conclusion here might be less representative for LBP patients from other locations as well as non-Chinese population.

## Conclusion

The outcomes of this research show that both EQ-5D-5 L and SF-6D are valid instruments in Chinese low back pain patients, but these two measures cannot generally be used interchangeably. The EQ-5D-5 L was superior to the SF-6D in Chinese low back pain patients attending this hospital, with stronger correlation to ODI, better known-groups validity. Further study needs to evaluate other factors, such as responsiveness and reliability.

## Abbreviations

AUC: Area under the curve
EQ-5D: EuroQol 5-dimension
EQ-5D-3 L: 3-level version of the EQ-5D
EQ-5D-5 L: 5-level EuroQol 5-dimension
EQ-VAS: EuroQol visual analog scale
ES: Effect size
HRQL: Health-related quality of life
ICCs: Intra-class correlation coefficients
LBP: Low back pain
MCS: Mental component summary
ODI: Oswestry Disability Index
PCS: Physical component summary
QALYs: Quality-adjusted life years
RE: Relative efficiency
ROC: Receiver operating characteristic
SD: Standard deviations
SF-36: 36-item Short Form Health Survey
SF-6D: Short Form 6-dimension
